# Zinc-Induced Copper Deficiency Myeloneuropathy Masquerading as Paraneoplastic Syndrome: A Case Report

**DOI:** 10.7759/cureus.82995

**Published:** 2025-04-25

**Authors:** Vladimir Osadchyi, Sarah N Van Antwerp, James Vredenburgh

**Affiliations:** 1 Internal Medicine, University of Connecticut, Farmington, USA; 2 Hematology and Oncology, Saint Francis Hospital and Medical Center, Hartford, USA

**Keywords:** copper deficiency, copper myeloneuropathy, hemato-oncology, neurological manifestations, paraneoplastic syndrome

## Abstract

Copper deficiency is a rare but reversible cause of myeloneuropathy, often overlooked in patients presenting with progressive neurological deficits. Excessive zinc intake, particularly from denture adhesives, can lead to copper depletion through competitive inhibition of intestinal absorption.

We present the case of a 63-year-old female with a history of chronic obstructive pulmonary disease (COPD), Stage IIIA triple-negative breast cancer (TNBC) (status post mastectomy with adjuvant therapy), peripheral neuropathy, and a prior cecal adenoma who was admitted for hematochezia and progressive generalized weakness over several weeks. Imaging revealed a suspicious 1.4 cm pulmonary nodule in the left lower lobe and a 2.6 cm x 1.8 cm x 1.5 cm destructive lytic lesion in the left parietal bone. Given her worsening lower extremity weakness, concern for a paraneoplastic syndrome prompted a lumbar puncture and paraneoplastic antibody panel, both of which were negative. The patient underwent a five-day course of intravenous immunoglobulin (IVIG) without improvement.

Further metabolic workup revealed profound copper deficiency of 417 µg/L (reference range: 810-1,990 µg/L). Upon further questioning, the patient reported chronic use of zinc-containing denture adhesive. She was initiated on copper supplementation with significant neurological improvement over several months, transitioning from being wheelchair bound to ambulating with a walker. Pathology from resection of the skull lesion revealed a World Health Organization (WHO) grade 1 meningioma, while the lung lesion was treated with CyberKnife, confirming Stage IA squamous cell carcinoma.

This case highlights the importance of considering nutritional deficiencies in patients with symptoms of progressive myeloneuropathy, such as gait disturbances, sensory ataxia, or spasticity, particularly in those with risk factors such as chronic denture adhesive use. Clinicians should maintain a high index of suspicion for copper deficiency in cases mimicking paraneoplastic syndromes.

## Introduction

Copper is an essential trace element that plays a crucial role in numerous physiological processes, including hematopoiesis, mitochondrial respiration, immune function, and maintenance of the nervous system [1,2]. It serves as a cofactor for several enzymes, including cytochrome c oxidase, superoxide dismutase, and ceruloplasmin, which are critical for oxidative phosphorylation, antioxidant defense, and iron metabolism, respectively [3,4].

Copper deficiency is an uncommon but clinically significant cause of neurological dysfunction, often presenting as progressive sensory ataxia, myelopathy, or peripheral neuropathy [5]. The neurological manifestations of copper deficiency closely resemble subacute combined degeneration of the spinal cord seen in vitamin B12 deficiency, with involvement of the dorsal columns and corticospinal tracts, leading to proprioceptive deficits, spasticity, and gait disturbances [6]. In some cases, copper deficiency can also contribute to cytopenias, particularly anemia and leukopenia, due to its role in iron homeostasis [7].

One underrecognized but well-documented cause of acquired copper deficiency is excessive zinc intake. Zinc competes with copper for absorption in the gastrointestinal tract by upregulating enterocyte metallothionein, a protein that binds copper with a higher affinity than zinc, leading to sequestration and eventual excretion of copper [8,9]. Chronic zinc exposure, even at doses not traditionally considered toxic, can lead to progressive copper depletion, resulting in neurological deterioration.

A particularly insidious source of zinc overexposure is prolonged use of zinc-containing denture adhesives, which may be applied in excessive amounts by patients unaware of their potential toxicity [10]. In 2011, the U.S. Food and Drug Administration (FDA) acknowledged reports linking excessive use of zinc-containing denture adhesives to adverse health effects, including nerve damage. Despite manufacturer warnings and subsequent reformulations to remove zinc from most commercially available denture creams, older formulations and excessive application can still pose a risk, especially in elderly patients or those with prolonged use [11].

This case highlights a rare but treatable cause of progressive neurological dysfunction due to zinc-induced copper deficiency in a patient initially suspected of having a paraneoplastic syndrome. Given the potential for irreversible neurological damage, clinicians must maintain a high index of suspicion for copper deficiency in patients with unexplained myelopathy, neuropathy, or cytopenias, particularly those with risk factors such as denture adhesive use.

## Case presentation

A 63-year-old female with a history of chronic obstructive pulmonary disease (COPD), tobacco use disorder, Stage IIIA triple-negative breast cancer (TNBC) (status post bilateral mastectomy with adjuvant chemotherapy and radiation), and peripheral neuropathy presented with hematochezia and progressive generalized weakness over several weeks to the point that she had become wheelchair bound.

**Figure 1:**
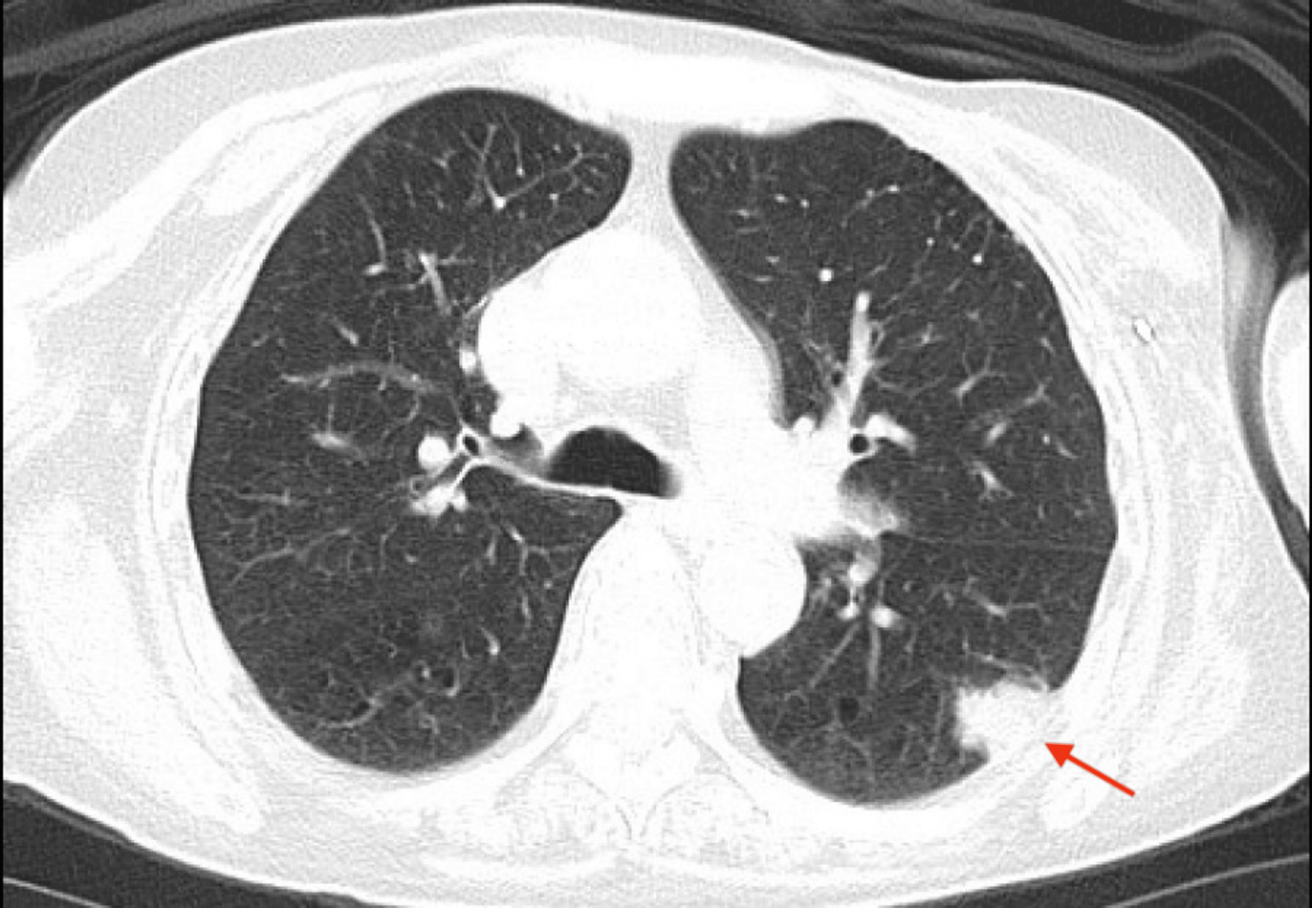
A computed tomography (CT) scan of the chest demonstrating a 1.9 cm left lower lobe pleural-based solid nodule as indicated by the red arrow.

**Figure 2:**
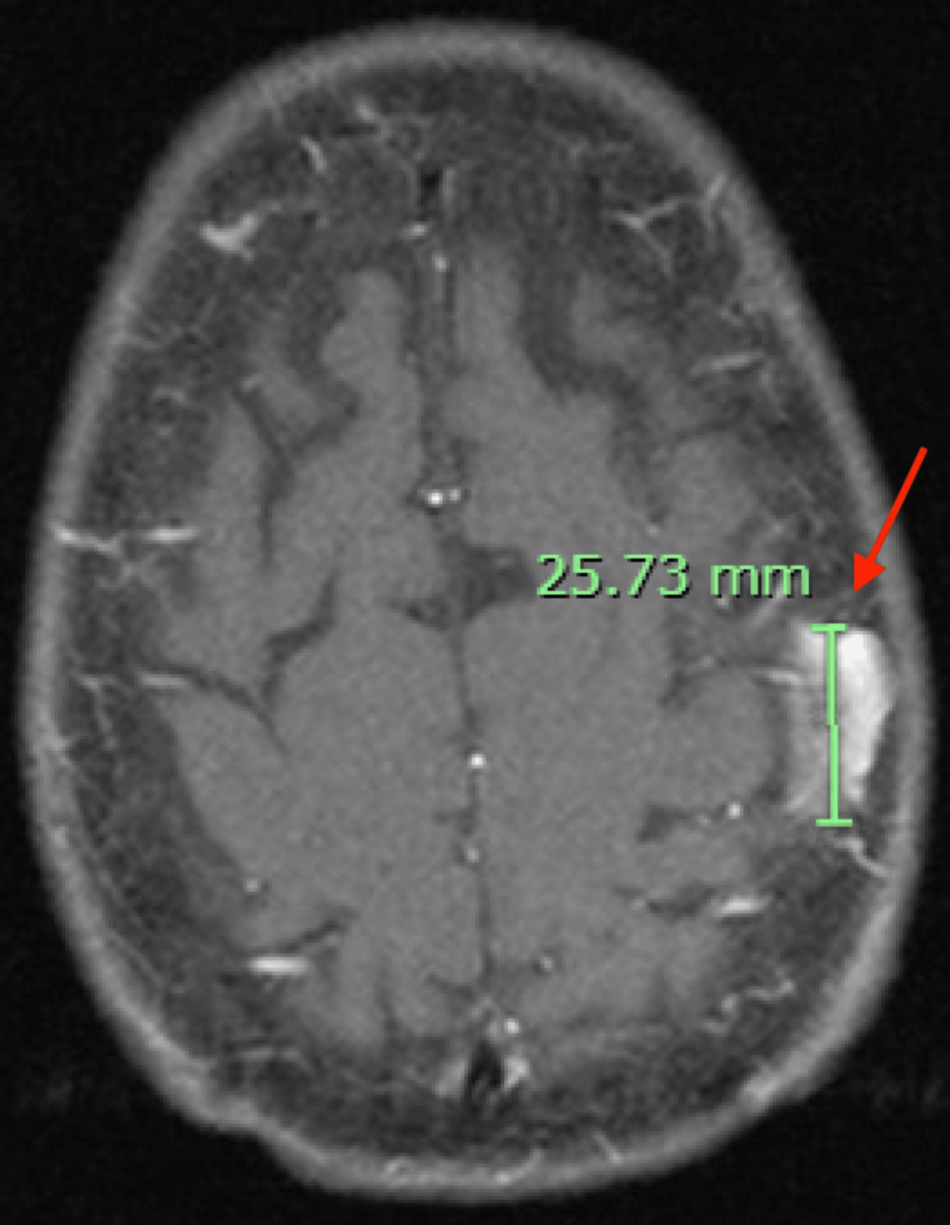
A computed tomography (CT) scan of the head demonstrating the left parietal skull mass extending from the inner table to the outer table calvarium, measuring 25.73 mm in the greatest length, as indicated by the red arrow. No intracranial extension is seen. No metastatic disease to the brain parenchyma is appreciated.

She had a known left lower lobe pleural-based lung nodule measuring 1.9 cm, previously identified on lung cancer screening and under evaluation for malignancy (Figure 1). The patient's neurological symptoms have been ongoing for approximately one year, but she noticed an acute worsening over several weeks during the patient became wheelchair-dependent. Due to this acuity, a computed tomography (CT) scan of the head was performed and revealed a 2.6 cm x 1.8 cm x 1.5 cm lytic lesion in the left parietal bone concerning for metastatic disease (Figure 2). A PET/CT scan demonstrated intense uptake in a 1.4 cm pleural-based nodule in the left lower lobe, while the left parietal bone lesion had mild tracer uptake. Given these findings, paraneoplastic syndrome in the context of presumed lung malignancy was strongly suspected, and the patient was admitted to the hospital for rapid initiation of intravenous immunoglobulin (IVIG) and to expedite the necessary workup. 

The patient had a prior diagnosis of chemotherapy-induced peripheral neuropathy with electromyography (EMG) demonstrating mild sensory axonal neuropathy in the lower extremities and left peroneal neuropathy at the fibular neck. However, her neurological symptoms had worsened significantly, prompting concern for a paraneoplastic etiology.

A lumbar puncture was performed, and the patient was initiated on an empiric five-day course of IVIG therapy (Gammagard 10%, 0.4 g/kg daily). Unfortunately, the patient did not experience any clinical improvement. After a few days, cerebrospinal fluid (CSF) cytology results were negative for malignant cells. Serum and CSF paraneoplastic antibody panels were also negative (Table 1).

**Table 1:** Serum paraneoplastic autoantibody panel with corresponding reference ranges. All tested autoantibodies were negative.

Paraneoplastic autoantibody (Serum)	Patient result	Reference range	Units
Amphiphysin antibody	Negative	Negative	-
AGNA-1 (Anti-glial nuclear antibody)	Negative	Negative	-
ANNA-1 (Anti-Hu)	Negative	Negative	-
ANNA-2 (Anti-Ri)	Negative	Negative	-
ANNA-3	Negative	Negative	-
CRMP-5-IgG	Negative	Negative	-
Neuronal voltage-gated potassium channel Ab	0.00	<0.02	nmol/L
P/Q-type calcium channel antibody	0.00	<0.02	nmol/L
PCA-1 (Anti-Yo)	Negative	Negative	-
PCA-2	Negative	Negative	-
PCA-Tr	Negative	Negative	-

Further metabolic workup revealed profound copper deficiency 417 µg/L (reference range: 810-1,990 µg/L) (Table 2). Upon additional history-taking, the patient endorsed frequent use of zinc-containing denture adhesive. Given the well-documented competitive inhibition of copper absorption by excessive zinc intake, her copper deficiency was suspected as the underlying cause of her progressive neurological decline.

**Table 2:** Serum laboratory findings for neurological evaluation. Serum laboratory testing was conducted as part of the evaluation for the patient's neurological symptoms. All results were within normal limits, except for copper levels, which were decreased.

Lab test	Patient value	Reference range	Units
Thiamine (Vitamin B1)	80	38-122	ug/L
Vitamin B6	7	5-50	ug/L
Vitamin B12	703	180-914	pg/mL
Vitamin E	1116	500-1,800	ug/dL
Thyroid-stimulating hormone (TSH)	3.91	0.45-5.33	uIU/mL
Folate	8.7	>3.0	ng/mL
Zinc	96	60-130	ug/dL
Copper	417	810-1,990	ug/L

The patient was initiated on copper supplementation with 2 mg of copper gluconate tablet daily, leading to significant neurological improvement over the following weeks and months. After approximately six months of supplementation and rigorous physical therapy, the patient was able to ambulate with a walker. Further evaluation of her presumed metastatic skull lesion revealed a WHO grade 1 meningioma rather than malignancy. Meanwhile, her pulmonary lesion was treated with CyberKnife radiotherapy and was ultimately staged as Stage IA squamous cell carcinoma.

## Discussion

Copper plays a fundamental role in nervous system function, hematopoiesis, and mitochondrial energy metabolism. It serves as a cofactor for several essential enzymes, including cytochrome c oxidase, ceruloplasmin, and superoxide dismutase, which are critical for oxidative metabolism, iron transport, and neuroprotection, respectively [1,2]. Copper deficiency can manifest with a wide array of clinical symptoms, primarily affecting the nervous system and hematologic function.

Neurologically, copper deficiency myeloneuropathy presents with progressive sensory ataxia, gait disturbances, and distal sensory loss, closely resembling subacute combined degeneration of the spinal cord seen in vitamin B12 deficiency [3,4]. The predominant involvement of the dorsal columns and corticospinal tracts results in proprioceptive deficits and spasticity, which may be mistaken for neurodegenerative conditions or paraneoplastic syndromes [5]. Additionally, peripheral neuropathy is common, with EMG often showing axonal sensorimotor neuropathy and features similar to chronic inflammatory demyelinating polyneuropathy (CIDP) [6]. Brain and spinal MRI in copper-deficient patients frequently reveal hyperintensities in the dorsal columns, similar to B12 deficiency-related myelopathy [7]. These radiologic similarities often lead to misdiagnosis, delaying appropriate intervention. Unlike vitamin B12 deficiency, however, copper deficiency is not associated with macrocytosis or elevated methylmalonic acid levels, providing a key differentiating feature [8]. Copper is critical for iron metabolism, and its deficiency often leads to anemia and neutropenia, mimicking myelodysplastic syndromes (MDS) [9]. Microcytic or normocytic anemia occurs due to impaired iron mobilization, resembling iron deficiency anemia, but it is refractory to iron supplementation [10]. Bone marrow biopsy in severe cases may show cytoplasmic vacuolization of erythroid and myeloid precursors, a hallmark finding of copper deficiency anemia [11]. Copper is absorbed in the proximal small intestine, and its homeostasis is tightly regulated by intestinal transporters. Zinc competes with copper absorption by inducing enterocyte metallothionein, a protein with a high affinity for copper. Once bound, copper is sequestered within enterocytes and subsequently lost during epithelial cell turnover, leading to progressive depletion [12].

Denture adhesives containing excessive zinc have been implicated in multiple cases of hypocupremia-associated myeloneuropathy, often with irreversible neurological damage if not identified early [13]. Nations et al. described a series of patients with severe neurological dysfunction linked to chronic denture cream use, highlighting the underrecognized risk of zinc toxicity in dental products [14]. Many commercial denture creams have since been reformulated to remove zinc, yet older formulations and excessive application continue to pose a health risk, particularly in elderly patients or those with prolonged denture use [15].

This case underscores the diagnostic challenges of copper deficiency myeloneuropathy, particularly in patients with suspected paraneoplastic neurological syndromes (PNS). PNS is an immune-mediated disorder that occurs in the setting of malignancy and frequently presents with progressive neurological dysfunction, sensory neuropathy, or cerebellar ataxia [16]. In our patient, the presence of a pulmonary nodule and skull lesion, coupled with her worsening neurological symptoms, raised concern for paraneoplastic syndrome. However, her negative CSF cytology and serum paraneoplastic antibody panel, along with a lack of improvement with intravenous immunoglobulin (IVIG) therapy, pointed toward an alternative etiology. The eventual discovery of profound copper deficiency (417 µg/L; reference range: 810-1,990 µg/L) was critical for the correct diagnosis and successful treatment.

Copper deficiency myeloneuropathy is potentially reversible, but early intervention is crucial to prevent permanent neurological damage. Oral or intravenous copper supplementation is the mainstay of treatment, with oral copper sulfate (2-4 mg/day) or intravenous copper (2 mg/day for 5-7 days, followed by oral therapy) commonly used in severe cases [17]. Neurological improvement is often gradual and incomplete, with sensory and motor deficits persisting for months despite copper repletion. Nations et al. reported that only 44% of patients had significant neurological recovery, emphasizing the importance of early recognition [14]. MRI abnormalities may take months to resolve, and follow-up imaging is recommended for monitoring progression [18].

Given the increasing awareness of trace metal imbalances, clinicians should consider routine screening for copper and zinc levels in patients with progressive myelopathy or neuropathy of unclear etiology. Additionally, healthcare providers should be aware of zinc-containing dental products as a potential source of chronic exposure, particularly in elderly populations or those with long-term denture use.

## Conclusions

This case highlights the importance of considering zinc-induced copper deficiency myeloneuropathy in patients presenting with progressive neurological decline, particularly when paraneoplastic syndromes or neurodegenerative diseases are suspected. The potential for misdiagnosis and delays in treatment can lead to irreversible neurological impairment, making early recognition essential.

A thorough supplement and dental product history is critical, as many patients unknowingly expose themselves to excess zinc through denture adhesives or other sources. This often-overlooked etiology requires heightened clinical awareness to prevent unnecessary diagnostic procedures, inappropriate treatments, and prolonged morbidity.

Most importantly, timely identification and copper repletion can lead to substantial neurological recovery, halting progression and preventing permanent disability. As in our case, the patient was able to transition from being wheelchair-dependent to ambulating with a walker after several months of copper supplementation. As awareness of trace metal imbalances grows, clinicians should actively screen for copper deficiency in unexplained myeloneuropathy, ensuring prompt intervention for a potentially reversible yet debilitating condition.
